# Impact of a Cellular Host-Response Sepsis Diagnostic on Clinical Decision Making in the Emergency Department: A Vignette-Based Study

**DOI:** 10.1016/j.acepjo.2026.100358

**Published:** 2026-03-28

**Authors:** Roya Sheybani, Hollis R. O’Neal, Christopher B. Thomas, Robert Scoggins, Christopher Pfaff, Howard Smithline, Donna M. Wolk, Eric Melnychuk, Tom P. Aufderheide, Thomas Carver, Matthew Chinn, Nathan A. Ledeboer, Chadd K. Kraus

**Affiliations:** 1 Cytovale, Inc, San Francisco, California, USA; 2 Our Lady of the Lake Regional Medical Center, Baton Rouge, Louisiana, USA; 3 Louisiana State University Health Sciences Center, New Orleans, Louisiana, USA; 4 Franciscan Missionaries of Our Lady Health System, Baton Rouge, Louisiana, USA; 5 Kootenai Health, Coeur d’Alene, Idaho, USA; 6 Department of Emergency Medicine, Baystate Medical Center, University of Massachusetts, Springfield, Massachusetts, USA; 7 Chan Medical School – Baystate, Springfield, Massachusetts, USA; 8 Geisinger Medical Center Danville, Danville, Pennsylvania, USA; 9 Department of Emergency Medicine, Medical College of Wisconsin, Milwaukee, Wisconsin, USA; 10 Division of Trauma and Critical Care Surgery, Medical College of Wisconsin, Milwaukee, Wisconsin, USA; 11 Department of Pathology and Laboratory Medicine, Medical College of Wisconsin, Milwaukee, Wisconsin, USA; 12 Department of Emergency and Hospital Medicine, at Lehigh Valley Health Network, Allentown, Pennsylvania, USA

**Keywords:** sepsis diagnosis, emergency department, decision impact, immune dysregulation, clinician confidence

## Abstract

**Objectives:**

Sepsis is a complex pathogen–host interaction that traditional laboratories do not easily detect. A test that rapidly assesses immune dysregulation could improve early diagnosis and clinical decision making. This study evaluates ease of application of a new host-response sepsis diagnostic test (IntelliSep; Cytovale, Inc) and its ability to affect decision making, risk stratification, and diagnostic efficacy in the emergency department (ED).

**Methods:**

This was a case vignette–based, randomized, multisite decision-impact study. We conducted the study with ED clinicians from 3 health care institutions, selected among the 5 participating institutions from a previous observational study. Participants were ED-attending physician/fellows, residents, and advanced practice clinicians. The study excluded interns. Finally, 13 clinicians at site 1, 28 at site 2, and 11 at site 3 (total, n = 52) were included. The study used retrospectively de-identified data from patient cases to generate 100 case vignettes. Each participant reviewed 10 cases with IntelliSep results and 10 cases without.

**Results:**

The study assessed outcomes including impact of the test results on clinical decision making, confidence in decision, and diagnostic accuracy. The test supported or changed/augmented clinician decisions in 86% of cases. The presence of the test significantly increased the proportion of raters with high confidence in their ratings (from 53.1% without, to 63.1% with). Additionally, diagnostic accuracy increased, with a notable increase in sensitivity for critical bands, from 73.1% without the test to 82.8% with it (95% CI, 63.5-81.3 vs 74.6-89.1); this increase was not statistically significant (*P* = .06).

**Conclusion:**

This paper-based study demonstrates that the IntelliSep host-response sepsis test results could impact clinician decisions and may improve the quality of clinical decision making.

**The Bottom Line**

Sepsis remains a challenging diagnosis in emergency departments, where rapid decision making is complicated by vague clinical presentations and conditions that closely mimic sepsis. This vignette-based decision-impact study asks whether a cellular host response–based sepsis risk-stratification tool can change how emergency department clinicians diagnose and treat patients with possible sepsis. In this modeled setting, clinicians reported that the test results supported or influenced decisions in 86% of cases and could lead to important changes in clinical pathways. Following brief training, the test may increase clinician confidence, improve diagnostic accuracy, and improve sepsis care in the emergency department.

## 1 Introduction

### 1.1 Background

Sepsis remains a leading cause of hospital-related mortality despite decades of research focused on improving diagnosis and treatment.^1^ Over 80% of sepsis cases present to the emergency department (ED), and rapid treatment is essential for optimizing outcomes.^2^^,^^3^ Part of the difficulty is that sepsis is a complex pathogen–host interaction that traditional clinical and laboratory evaluations do not easily detect.^4^ A new class of US Food and Drug Administration–cleared host-response sepsis diagnostics (HRSD) promises to reveal potentially septic patients early in their ED course. One such test, IntelliSep (Cytovale, Inc), uses white blood cell biomechanics to provide a signal about immune dysregulation and produces a score stratified into 3 readily interpretable bands of increasing likelihood of sepsis.^5^

### 1.2 Importance

It is unclear whether integration of such a test into ED workflows will influence clinical decisions and patient outcomes. The clinical utility of a diagnostic test depends on how well it fits existing workflows, while providing results that are easily interpreted, are relevant, and can positively impact clinical outcomes. This is partially determined by how readily clinicians can apply it in routine practice to drive actions that benefit the patient. Previous work has shown that decision-impact studies can provide valuable insights regarding the utility of diagnostics.^6^

### 1.3 Goals of this investigation

The purpose of this study is to evaluate the ease of application and potential impact of an HRSD on the decision making and diagnostic performance of ED clinicians in a modeled environment.

## 2 Methods

### 2.1 Study Design

We conducted a case vignette–based, randomized, multisite decision impact study designed to assess how a new HRSD, the IntelliSep test, could influence clinician decision making and diagnostic accuracy in sepsis care.

### 2.2 Setting

We conducted the study with ED clinicians from 3 health care institutions—site 1 (S1): Springfield, Massachusetts; site 2 (S2): Milwaukee, Wisconsin; and site 3 (S3): Danville, Pennsylvania (details included in Table S1). We selected these sites from among the 5 institutions that participated in the earlier observational study.

### 2.3 Selection of Participants

ED clinicians volunteered for the project, were chosen by the study leadership at each site, and were compensated for their time. Eligible participants included teaching and nonteaching attending physicians and emergency medicine resident physicians in the postgraduate year 2 or higher, physicians in fellowship training, and advanced practice clinicians; the study excluded interns due to insufficient experience and expertise in emergency medicine. At S1, 13 clinicians participated (10 fellows/attendings, 2 residents, and 1 PA); at S2, 28 clinicians participated (12 fellows/attendings, 14 residents, and 2 physician assistants); and at S3, 11 clinicians participated (all fellows/attendings) (Table 1).

**Table 1:** Credentials and level of training for participating clinicians at each site.

Credentials	Level of training	Years of experience	Site 1 (S1) Springfield, Massachusetts	Site 2 (S2), Milwaukee, Wisconsin	Site 3 (S3) Danville, Pennsylvania
MD/DO	Faculty/attending	>10	3	6	4
		5-10	4	0	5
		<5	2	6	2
	Resident		3	14	0
PA	—	—	1	2	0

### 2.4 Vignette Preparation

We prospectively collected clinical cases from a pragmatic multicenter, nonregistrational observational study, to be published separately. In that study, investigators explored different implementations of the HRSD as part of the ED sepsis protocol. We collected the cases from routine emergency care encounters at 5 geographically diverse EDs (in Massachusetts, Wisconsin, Pennsylvania, Louisiana, and Idaho) (Table S1). Owing to time constraints, we used data from 4 of the institutions to develop case vignettes.

A retrospective adjudication process, blinded to the test results, established a reference standard to evaluate diagnostic performance and yielded comparable rates of sepsis (per the Sepsis-3 criteria) across the pool of vignettes. Briefly, to be considered positive for sepsis, patients must have had each of the following 3 components: (1) infection (present on presentation to the ED), (2) organ dysfunction (manifesting within 3 days of the ED visit), and (3) organ dysfunction caused by a dysregulated host response to the infection. We used a rigorous 2-tiered process to adjudicate each component. First, study coordinators extracted data from the electronic health record to complete an objective evaluation for infection (reference standard)^7^ and organ dysfunction (reference standard).^8^ We considered patients to be disease state positive (ie, septic) when infection was present and the maximum Sequential Organ Failure Assessment (SOFA) score calculated over the first 3 days following presentation was 2 or more points over baseline. Subsequently, for a subset of cases (comprising at least 25% of the cases at each enrollment site), a site investigator with access to the medical record, but blinded to the IntelliSep test results, completed a structured adjudication process and recorded pertinent clinical information, at least 15 days after the patient presentation to the ED. In all cases reviewed by this methodology, we used the physician assessment as the final disease state. The adjudication of these cases for sepsis allowed us to assess whether the test may improve diagnostic accuracy for the case vignettes.

The study retrospectively used de-identified data to generate 100 case vignettes. The vignette preparation process is detailed in Figure S1. For each of the 4 sites, 25 case vignettes were chosen at random from the pool of potential vignettes (ie, all enrolled cases for which both disease state and the IntelliSep test result were known; n = 689 in total across the 4 sites). We excluded cases in which the sepsis disease state was adjudicated as indeterminate (n = 7 of 696 cases; 1%). The selection process for vignettes was blinded to the concordance or discordance between the HRSD result and adjudicated sepsis. As such, vignettes included cases where the result was discordant with adjudicated sepsis status. For each site, the number of cases needed within each band was determined based on the distribution of patients across the test’s interpretation bands. We then used a random generator to select a subset of cases from those that fell within the interpretation band. This process ensured the ultimate set of case vignettes represented the distribution of cases across the test’s interpretation bands and within each band. The resulting subset from each site also represented the overall prevalence of disease from that site. Separately, we implemented branching logic in the electronic data capture system to enable the sepsis test result to be hidden during the first round of data collection and revealed during the second.

The WCG Institutional Review Board (IRB# 20240939) approved this study. Clinician responses were collected using the REDCap (Research Electronic Data Capture) system, a secure, web-based software platform.

### 2.5 Training

Prior to exposure to clinical case vignettes, participating clinicians completed a 3-part training. First, participants attended a 45-minute virtual study initiation session that a study member led, covering study setup and the new host-response sepsis test background. Following this, participants read the study protocol and watched a 23-minute educational video describing how the test works, its performance characteristics, and potential clinical interpretation. The training provided participants with critical information about the clinical accuracy of the test, including negative predictive value, positive predictive value, sensitivity, specificity, and likelihood ratios for each band of the test results as provided in published literature.^5^

### 2.6 Interventions and Measurements

Following training, participants received a randomly selected set of 10 case vignettes (from the pool of 100 prepared case vignettes), delivered as surveys through the REDCap system.^9^^,^^10^ An example case vignette has been provided in the Supplementary information. Each vignette included data available early in the patient’s ED presentation (eg, vital signs, chief complaint, demographics, comorbidities, baseline SOFA scores, ED history and physical examination notes, review of systems and examination findings, and results of routine tests such as chemistry, complete blood counts, urinalysis, lactate, and troponin, when available). For each vignette, the clinician entered their name, credentials, and level of training. Clinicians were asked to indicate their diagnosis (sepsis, infection, or other), rate their confidence in that diagnosis, and indicate whether they would expedite the SEP-1 care bundle for the patient.^11^ Participants were asked additional questions related to the use of sepsis-associated resources (eg, blood cultures, antibiotics, fluids, and vasoactive medications), but these results were not included in this analysis. We hid the HRSD test results from participants at this stage. As the number of participants was larger than the number of cases, each case was assigned to multiple participants.

**Figure 1:**
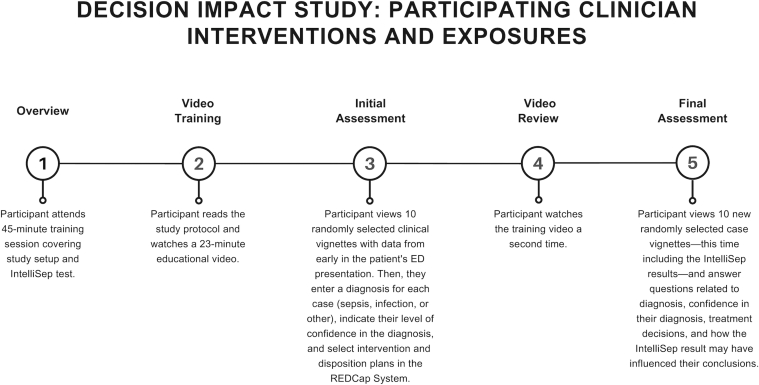
Overview of study participant workflow and exposures. This diagram illustrates the presentation of clinical vignettes, test exposure, and data collection phases used to assess the impact of a new host-response sepsis test on clinical decision-making.

After completing the first set of surveys, we asked participants to watch the HRSD educational video again, this time with a focus on the interpretation of results. Participants received a second set of 10 vignettes (from the pool of 100 prepared case vignettes) that included the HRSD test result. These cases were also assigned at random and may or may not have included vignettes seen during the first round. Participants answered the same group of questions from the first round. In addition, they were asked to indicate how the test results may have influenced their conclusions in this round. Vignettes could be reviewed by multiple participating physicians who were randomly assigned to see each case either with or without the test results. As a result, different clinicians could evaluate the same case under both conditions. Figure 1 shows participating clinician intervention and exposure steps. The vignette assignment process is detailed in Figure S2.

### 2.7 Outcomes

We defined the primary outcome as the impact of the HRSD test results on clinical decision making. Secondary outcomes included confidence in the decision and diagnostic accuracy based on the test results.

### 2.8 Data Analyses

We performed data analysis using Python Language Reference, version 3.9.7 (Python Software Foundation). Unless otherwise stated, we derived *P* values from nonparametric Mann–Whitney *U* test for continuous variables and the χ^2^ proportions test to test the differences in populations for binary and categorical data. We present descriptive statistics as means, SDs, medians, and first and third quartiles (Q1-Q3) for continuous variables and as counts and percentages for categorical variables. We used an α level of 5% for all analyses, unless otherwise stated. We did not perform data imputation; data were presented as recorded in the cases. Two-sided CIs for proportions are provided using the Clopper–Pearson method, where appropriate. We used McNemar test with the χ^2^ approximation to compare diagnostic performance with and without the host-response sepsis test results.

## 3 Results

### 3.1 Clinical Vignette Patient demographics

We collected a total of 1,040 completed case vignettes: 520 without the test results and 520 with the test results. Patient demographics, clinical characteristics, and test results featured in the vignettes were comparable between groups. We observed no statistically significant differences in age, sex, race, infection or sepsis status, SOFA scores, hospital admission rates, or blood culture results (Table 2).

**Table 2:** Patient demographics, characteristics, and testing results featured in the case vignettes.

Category	Subcategory	Adjudication		*P*
		Without test result (n = 520)	With test result (n = 520)	
Age (y)	Median (Q1-Q3)	69 (45-77)	65 (43-75)	.26
	Subjects ≥ 65 y	288 (55.4)	268 (51.5)	.24
Sex	Male	270 (51.9)	276 (53.1)	.76
	Female	250 (48.1)	244 (46.9)	.76
Race	Black or African American	110 (21.2)	115 (22.1)	.79
	White	383 (73.7)	380 (73.1)	.79
	Other	27 (5.2)	25 (4.8)	.79
Infected, by adjudication	Yes	261 (50.2)	276 (53.1)	.48
Sepsis, by adjudication	Yes	167 (32.1)	179 (34.4)	.47
Sepsis, by adjudication, per ISI band population, n of band (% of band)	Interpretation Band 1	19 of 253 (7.5)	16 of 213 (7.5)	1.00
	Interpretation Band 2	63 of 144 (43.8)	63 of 150 (42.0)	.85
	Interpretation Band 3	85 of 123 (69.1)	100 of 157 (63.7)	.41
Cumulative 30-d in-hospital mortality	All-cause	32 (6.2)	31 (6.0)	1.00
	Sepsis associated	26 (5.0)	26 (5.0)	1.00
SOFA, 3-d maximum (baseline subtracted)	Median (Q1-Q3)	2 (0-5)	2 (1-4)	.68
Admitted to hospital	Yes	445 (85.6)	447 (86.0)	.93
Blood culture	Number ordered	367 (70.6)	348 (66.9)	.23
	Number positive (of ordered)	125 (34.0)	118 (33.9)	.34
	Number positive (of total)	125 (24.0)	118 (22.7)	.6
Blood culture positive per ISI band population, n of band (% of band)	Interpretation Band 1	39 of 253 (15.5)	29 of 213 (13.6)	.68
	Interpretation Band 2	52 of 144 (36.1)	44 of 150 (29.3)	.27
	Interpretation Band 3	34 of 123 (27.6)	45 of 157 (28.7)	.96

ISI band, IntelliSep test interpretation band. Values are n (%) unless specified.

### 3.2 Participating Clinician-Decision Support

**Figure 2:**
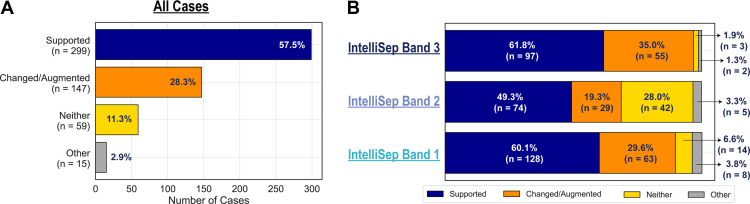
Perceived impact of receiving the IntelliSep test results on participating clinician decision-making (A) overall and by (B) test Interpretation Band.

The sepsis test supported or changed/augmented participating clinician decisions in 86% of cases. Of these, 58% reported that the test results supported their initial decision, while 28% said the sepsis test result led them to change it. The remaining 14% noted the test did not impact their decision making (rationale in Table S2). The impact varied by interpretation band: band 3 results (which indicate high risk of sepsis) had the greatest perceived impact: in 97% of band 3 cases, clinicians reported the sepsis test result informed or influenced their decision-making process, with 35% resulting in a change in decision. Band 2 results had the least impact, affecting decision making in 69% of clinician decisions (Fig 2). Participants who indicated that the test neither supported nor changed their decisions provided notes (Table S2).

### 3.3 Impact on Participating Clinician Confidence

**Figure 3:**
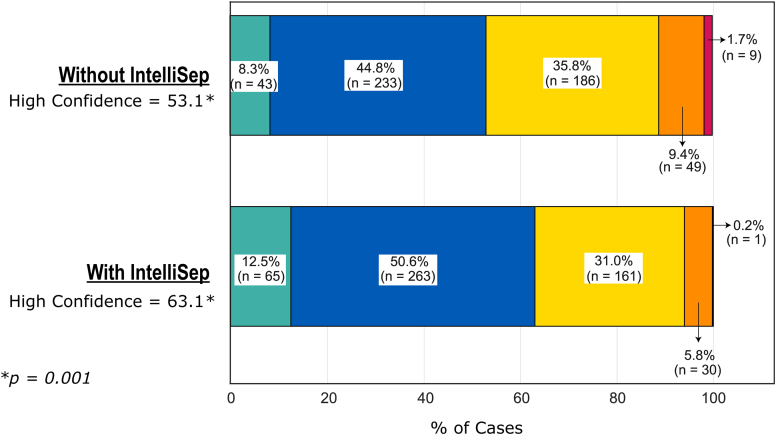
Impact of the IntelliSep test results on clinician confidence in their diagnosis.

**Figure 4:**
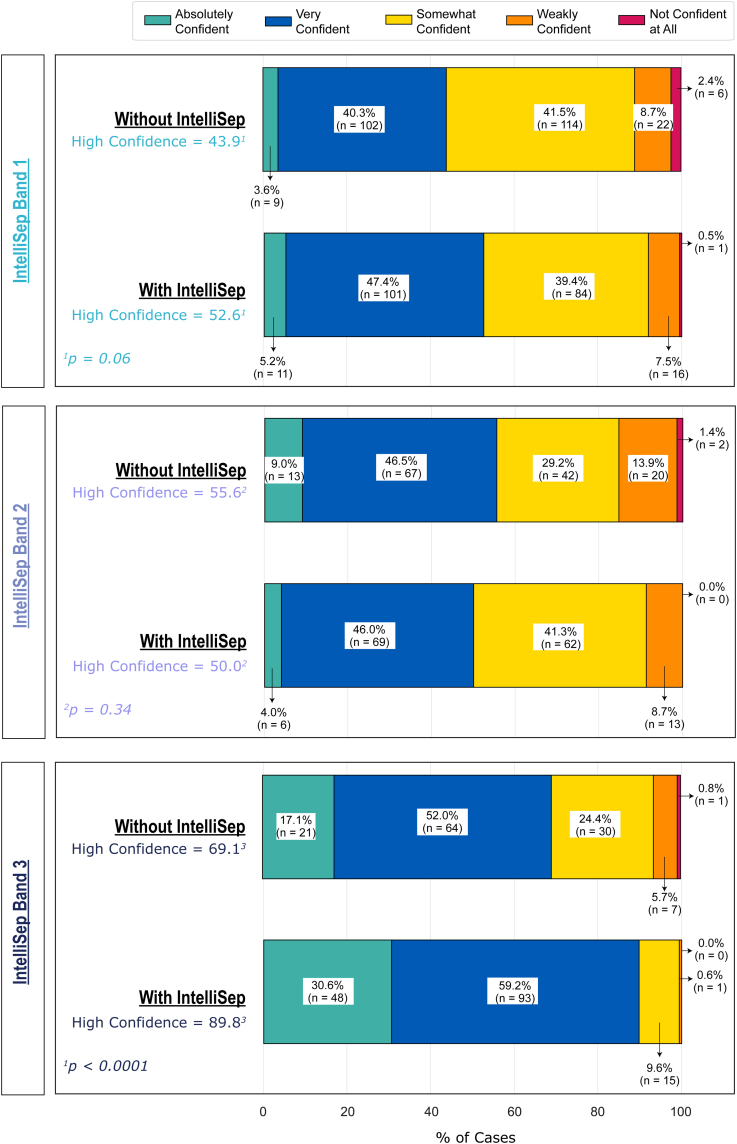
Impact of the IntelliSep test results on clinician confidence in their diagnosis by test Interpretation Band.

Clinician confidence in diagnostic decisions increased after seeing the sepsis test results, with a notable shift from lower (somewhat/weakly confident) to higher (absolutely/very confident) confidence levels. Overall, 63.1% of clinicians demonstrated high confidence (combining absolutely and very confident), compared with 53.1% reporting high confidence without the sepsis test available, with a statistically significant increase (*P* < .01) (Fig 3). The distribution of clinician confidence by band, with and without sepsis test results, is presented in Figure 4.

### 3.4 Impact on Diagnosis

Availability of sepsis test results influenced how participating clinicians classified sepsis in band 1 and band 3 vignette cases. Clinicians were less likely to classify band 1 patients as septic and more likely to classify band 3 patients as septic after viewing the HRSD results. However, there was no significant difference in the prevalence of sepsis within each band between the with- and without-sepsis test result groups (Table 2). Clinicians were also less likely to recommend expediting care to meet SEP-1 bundle 3-hour requirements for tier 1 (64% relative reduction; *P* < .001 respectively) but more likely for tier 3 patients (26% relative increase; *P* < .001). Notably, there was no significant difference in blood culture positivity within test bands between the with– and without–test result conditions (Table 2). We observed no significant differences in participating clinician behavior with regard to band 2 cases between the with– and without–test result conditions.

**Figure 5:**
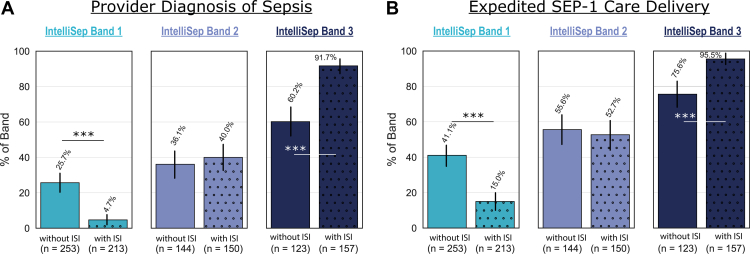
Impact of the IntelliSep test results on (A) clinician diagnosis of sepsis, and (B) expedited SEP-1 delivery. ∗∗∗*P* < .001. Error bars represent 95% CI.

Diagnostic performance of participating clinicians changed with the addition of sepsis test results, particularly in cases with a band 1 (low probability) or band 3 (high probability) score. In band 1 cases, septic classifications decreased from 26% to 5% (*P* < .001), while in band 3 cases, septic classifications increased from 60% to 92% (*P* < .001) (Fig 5, Table 3).

**Table 3:** Diagnostic performance with and without host-response sepsis test results.

	Band	Without results (%)	With results (%)	Decision supported/augmented by test results
Clinician septic classification	1	25.7	4.7	—
	3	60.2	91.7	—
Positive percent agreement (sensitivity; %), value (95% CI)	1 and 3	73.1 (63.5-81.3)	82.8 (74.6-89.1)	86.9 (79.0-92.7)
	All Bands	64.1 (56.3-71.3)	70.4 (63.1-77.0)	80.0 (72.7-86.1)
Negative percent agreement (specificity; %), value (95% CI)	1 and 3	76.8 (66.6-83.8)	77.2 (68.0-84.1)	77.1 (67.5-84.3)
	All Bands	76.2 (68.8-82.3)	74.2 (66.7-80.0)	73.6 (65.5-80.2)

Since cases had been adjudicated for sepsis as part of the previous observational study, it was possible to compare each participant’s sepsis assessment with the original study’s reference standard, both for cases where the test result was available and those where it was not. The sensitivity (positive percent agreement with the adjudicated reference) achieved by clinicians with the result was notably better but not statistically significant (*P* = .06), while the specificity (negative percent agreement) was similar (*P* = .76) in both cases (Table 3). Importantly, cases where clinicians reported that the sepsis test result had supported or augmented their decision achieved greater sensitivity relative to those who did not have access to the results. Primary values reported (Table 3) are based on band 1 and band 3 cases; values in parentheses reflect results across all bands.

## 3 Limitations

Decision-impact testing evaluates the potential impact on clinical decision making. In assessing this impact, we presented raters with a set of cases with the host-response test result and cases without this result. Importantly, each rater reviewed a randomized set of cases, some with test results and some without. This approach was implemented to prevent clinical anchoring bias from impacting the consideration of the cases. While it may help limit anchoring bias, it hinders the ability of the study to show the direct impact of the IntelliSep test given that each participant did not necessarily review the same cases both with and without the test results. Rather, each case was presented multiple times with and without the test result to different participants, and the results were aggregated. While this provides a comparison of how the population may consider the cases with and without the test results, the direct impact of the test on an individual participant may be compromised.

As this was a paper-based study using simulated clinical case vignettes, participating clinicians may have had a different attitude toward diagnostic or therapeutic risks than in actual practice. In this study, we focused only on sepsis-associated diagnosis; we did not assess the costs and impacts associated with alternative diagnoses or diagnostic procedures. In addition, although the training was intended to provide background helpful for understanding the project, it may have introduced bias when making diagnostic and treatment decisions. Selection of clinicians at each site did not occur at random, and ordering effects could have led to some bias given the study design. This study focused on application within the ED context; the potential impact of the test in other settings, such as the intensive care unit or hospital inpatient units, is unclear.

## 4 Discussion

Sepsis remains a challenging diagnosis in EDs, where rapid, accurate decision making is complicated by vague clinical presentations and critical conditions that closely mimic sepsis. This evaluation clearly demonstrates the ease of participating clinicians using the IntelliSep host-response sepsis diagnostic test to impact clinical decision making and clinician confidence. Participating clinicians indicated that the test substantially influenced their clinical decisions, a result consistent with previous findings, demonstrating the test’s strong negative predictive value for band 1 and good positive predictive value for band 3.^5^ Given the urgency inherent in sepsis management, this underscores the value of the IntelliSep test in reducing diagnostic uncertainty.

Band 3 scores had a greater impact on diagnostic decisions than band 1, which may reflect clinicians’ tendency toward risk mitigation.^12^ Clinicians naturally err on the safe side when diagnosing potentially life-threatening conditions like sepsis, prompting more decisive action in response to higher-risk scores. Additionally, with the test result available, clinician diagnosis of sepsis more closely matched the adjudicated outcome, suggesting that using the IntelliSep test could lead to more accurate diagnoses.

ED clinicians need practical, actionable diagnostics to facilitate the right care, to the right patient, at the right time. This decision-impact study highlights the simplicity and ease of use of the test results, as demonstrated by clinicians applying them confidently after brief video training. Unlike diagnostic platforms requiring considerable onboarding or difficult alignment with ED workflows, the novel sepsis test delivers rapid results with straightforward interpretation easily integrated into protocols for adults presenting with signs and symptoms of infection—the same context used to develop the case vignettes. This usability makes it easier to design clinical pathways that support confident decision making. Compared with other host-response diagnostics, which may involve complex interpretation and require extensive training, the IntelliSep test appears practical to implement in real-time clinical settings.

Interventional studies are needed to determine how the test may impact patient care and validate these results. Future research should also assess how increased clinician confidence and diagnostic accuracy could directly affect patient outcomes, ED throughput, costs associated with sepsis bundle compliance, and antibiotic stewardship in prospective, real-world clinical settings.

In conclusion, this study demonstrates that the IntelliSep test could both impact clinician decisions and improve clinical decision-making quality. Importantly, successful application of the IntelliSep test required only minimal training. These findings suggest the test could be a practical but powerful tool for enhancing sepsis care in the ED.

## Author Contributions

Roya Sheybani: conceptualization, data collection, data analysis, critical review and evaluation of results, project administration, writing, review and editing of the paper, and study supervision. Hollis R. O’Neal and Christopher B. Thomas: conceptualization, methodology, study supervision, and review and editing of the paper. Robert Scoggins: conceptualization, study supervision, and review and editing of the paper. Christopher Pfaff: critical review and evaluation of results and writing, review, and editing of the paper. Howard Smithline, Donna M. Wolk, Eric Melnychuk, Tom P. Aufderheide, Thomas Carver, Matthew Chinn, Nathan A. Ledeboer, and Chadd K. Kraus, critical review and evaluation of results and review and editing of the paper.

## Funding and Support

Cytovale, Inc, funded this research, in part through Federal funds from the Department of Health and Human Services; Office of the Assistant Secretary for Preparedness and Response; and Biomedical Advanced Research and Development Authority (under Contract No. 75A50123C00066).

## Conflict of Interest

Roya Sheybani: employee of Cytovale, Inc. Hollis R. O’Neal and Christopher B. Thomas: received personal consulting fees from Cytovale, Inc. Robert Scoggins: affiliated with Cytovale, Inc, and holds equity in the company whose device is the subject of this study. Christopher Pfaff: employee of Cytovale, Inc. Howard Smithline: reports no conflicts of interest. Donna M. Wolk: reports research funding outside the submitted work from GenMark and Cepheid for diagnostic accuracy studies, including those related to evidence-based guidelines. Eric Melnychuk: editor for *JACEP Open*. Tom P. Aufderheide: reports no conflicts of interest; Thomas Carver: holds stock options for CLR Medical and received payments from Cytovale for medical monitoring and consulting. Matthew Chinn: reports no conflicts of interest. Nathan A. Ledeboer: holds stock options in Cytovale, Inc, and Inflammatix. Chadd K. Kraus: Senior Editor for *JACEP Open* and has previously received research consulting honoraria from Cytovale, Inc.
