# Supplementary material for: Impact of a Cellular Host-Response Sepsis Diagnostic on Clinical Decision Making in the Emergency Department: A Vignette-Based Study
Source: J Am Coll Emerg Physicians Open. 2026 Mar 28;7(3):100358. doi: 10.1016/j.acepjo.2026.100358 (PMC13054420; doi:10.1016/j.acepjo.2026.100358)

**Impact of a Cellular Host-Response Sepsis Diagnostic on Clinical Decision-Making in the Emergency Department: A Vignette-Based Study**

***Supplementary Information***

***Table of Contents***

[**Supplementary Table 1:** 2](#_Toc220606692)

[**Supplementary Figure 1:** 3](#_Toc220606693)

[**Supplementary Figure 2:** 4](#_Toc220606694)

[**Supplementary Table 2:** 5](#_Toc220606695)

[**Example Case Vignette** 8](#_Toc220606696)

**Supplementary Table 1:** Details of clinical sites from which the cases were collected (SQuISH-IER) and the sites from which study participants were recruited (SQuISH-DI).

| Study | Dates | Sites | IRB Approval |
| --- | --- | --- | --- |
| SQuISH-IER | Enrollment Dates:  Feb. 2023 – May 2024 | (1) University of Massachusetts Chan Medical School – Baystate, Springfield, Massachusetts, USA  (2) The Froedtert & the Medical College of Wisconsin, Milwaukee, WI, USA  (3) Geisinger Medical Center Danville, Danville, Pennsylvania, USA  (4) Our Lady of the Lake Regional Medical Center, Baton Rouge, Louisiana, USA  (5) Kootenai Health, Coeur d’Alene, Idaho, USA | Baystate Health IRB #BH-22-178 (exemption 10/19/2022)  MCW IRB #PRO00044966 (approved 1/28/2023)  GIRB #2023-1543 (approved 11/14/2023)  LSUHSC-NO IRB #4824 (approved 1/5/2023)  WCG IRB #20232327 (approved 5/22/2023) |
| SQuISH-DI | Apr. 2024 – Dec. 2024 | (1) University of Massachusetts Chan Medical School – Baystate, Springfield, Massachusetts, USA  (2) The Froedtert & the Medical College of Wisconsin, Milwaukee, WI, USA  (3) Geisinger Medical Center Danville, Danville, Pennsylvania, USA | WCG IRB #20240939 (approved 3/4/2024) |

**Supplementary Figure 1:** Flow chart showing the details of how vignettes were prepared using de-identified data collected as part of a multi-center observational study.
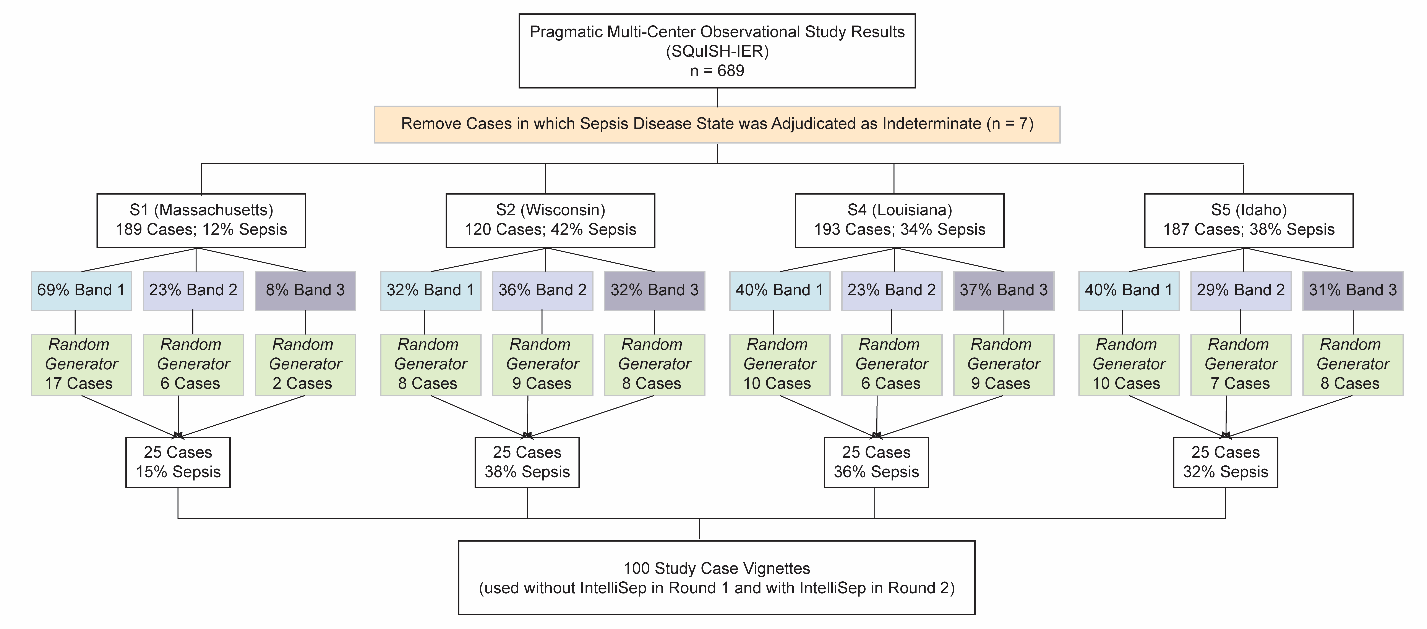
.

**Supplementary Figure 2:** *Flow chart showing the details of how case vignettes were assigned to study participants.*

*
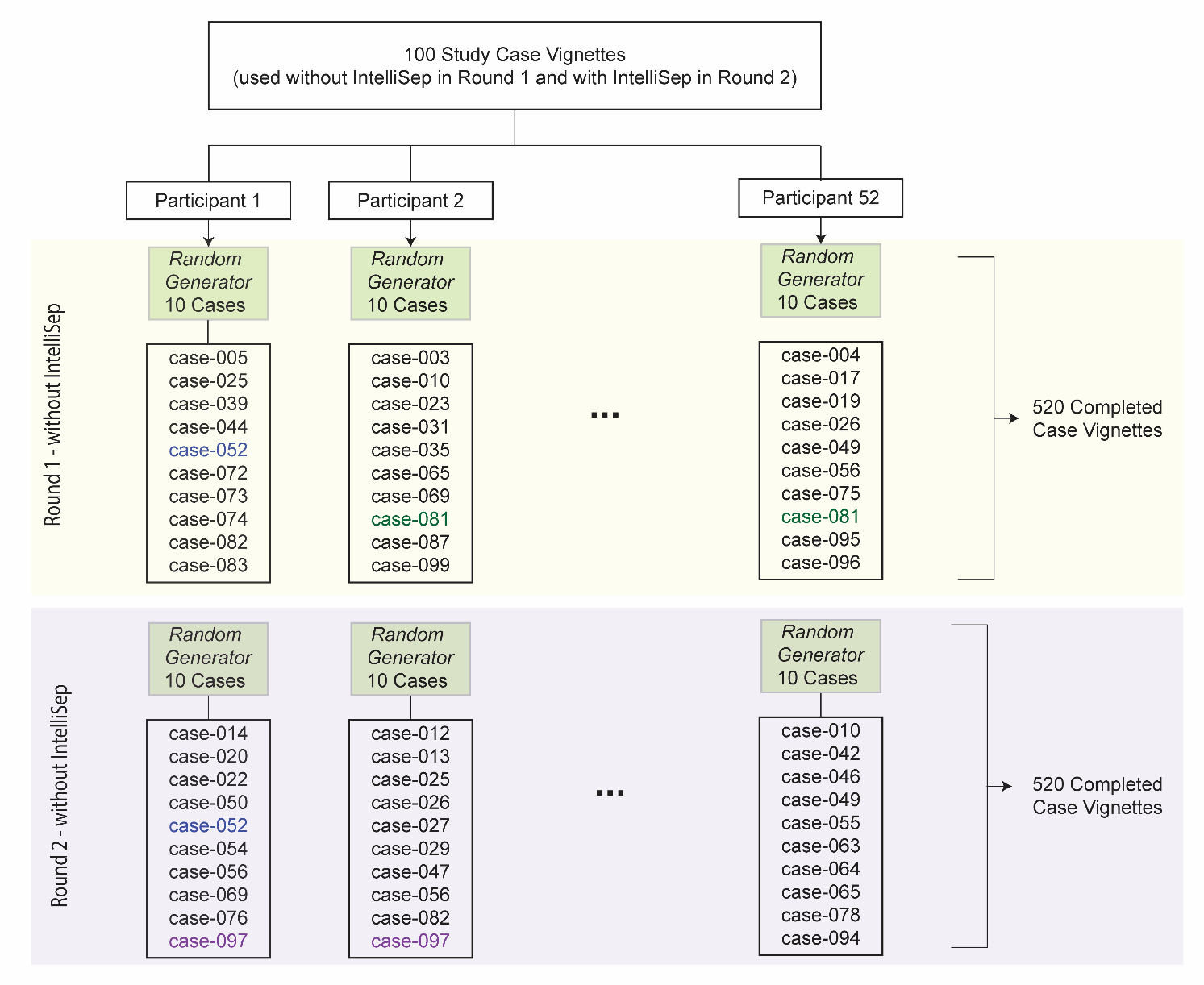
*

**Supplementary Table 2:** Notes provided by survey participants when then deemed the IntelliSep test results neither supported nor augmented their decisions.

| **Patient ID** | **IntelliSep Index (ISI) &**  **Interpretation Band** | **Retrospective Adjudication of Disease Status** | **Assessment by Participating Clinician** | | |
| --- | --- | --- | --- | --- | --- |
|  |  |  | **Assessment of Disease Status** | **Assessment of Impact of IntelliSep on their Decision** | **Notes** |
| 009-S2-105 | 1.5 / Band 1 | Infection/Not Sepsis | Sepsis | Other | LG: Questioned if IV antibiotics were required in ED. However, pt did meets SIRs criteria so treated empirically |
| 009-S1-170 | 2.9 / Band 1 | Not Infected | Other: CHF exacerbation, acute respiratory failure | Other | TP: Not sure I would have ordered the sepsis test in this case in the first place, but it does confirm my clinical impression |
| 009-S4-101 | 3.1 / Band 1 | Not Infected | Sepsis | Other | CS: Contradicted. Pre-test probability for sepsis is so high in a patient with treatment resistant pneumonia, should get admitted for IV antibiotics regardless. |
| 009-S1-025 | 3.7 / Band 1 | Not Infected | Other: Hypovolemic hyponatremia | Other | ER: Made me more concerned of missing infection, but didn't change my treatment or diagnostics. |
| 009-S5-143 | 3.7 / Band 1 | Sepsis | Other: sepsis or PE | Other | RV: It actually made me consider sepsis much less. I am not sure what the ddx would be though, but i continue to be concerned about this patient’s clinical course. I would treat for sepsis, until I have another alternative cause. |
| 009-S4-195 | 4.0 / Band 1 | Not Infected | Other: DVT, PE | Other | CB: **It did support my choice to rule out sepsis**. But I wanted to note that I ordered an ultrasound and there was no extremity option. And I would do a lower extremity ultrasound not a spine. |
| 009-S2-033 | 4.6 / Band 1 | Not Infected | Infection/Not Sepsis | Other | AP: Contrary to my hypothesis will ignore. |
| 009-S4-097 | 4.9 / Band 1 | Infection/Not Sepsis | Other: symptomatic bradycardia | Other | RV: This is a clinical case where infection could certainly be considered but it seems less likely. **The sepsis test confirmed my low suspicion, but also helps me see the pt is still critically ill and needs admission** |
| 009-S4-009 | 5.3 / Band 2 | Sepsis | Other: Bowel obstruction | Other | LG: Question possible infection; immunosuppression / hx HIV may be suppressing white count. |
| 009-S1-155 | 6.0 / Band 2 | Not Infected | Sepsis | Other | RV: I think that the score of Band 2 makes it challenging to pin point the etiology of the pt symptoms. I think with the overall picture, including the pts tachypnea, history, and also the UA, sepsis would be my largest concern. |
| 009-S2-086 | 6.0 / Band 2 | Sepsis | Other: GI bleed, dehydration, PE | Other | SG: I don't think it changed my diagnostic decisions but my treatment decisions. |
| 009-S4-187 | 6.0/Band 2 | Sepsis | Infection/Not Sepsis | Other | NM: decreased confidence |
| 009-S5-138 | 6.0/Band 2 | Not Infected | Sepsis | Other | SS: **Changed/Augmented**: would not have used a band 1 result as reason to not get cultures, ct (alp elev), as it has not been studied in transplant patients. However, I do respect the level 2 result and would not send home based on this. |
| 009-S1-034 | 6.3 / Band 3 | Sepsis | Infection/Not Sepsis | Other | EG: Did not play a role. |
| 009-S4-082 | 7.6 / Band 3 | Infection/Not Sepsis | Sepsis | Other | RV: **Strongly supported**. this sounds like a case of urosepsis, so I feel like its concerning for that. Also intrabdominal infection. The patient clearly is critically ill which the sepsis test lines up with. |
| 009-S1-181 | 0.1 / Band 1 | Not Infected | Sepsis | Neither | RB: This patient meets SIRS criteria so to me they warrant sepsis care even though they are Band 1. |
| 009-S1-181 | 0.1 / Band 1 | Not Infected | Infection/Not Sepsis | Neither | MA: Despite Intellisept results, do believe patient's clinical scenario represents infection and possible sepsis. Has fever, bandemia, oxygen requirement. |
| 009-S4-042 | 2.1 / Band 1 | Not Infected | Other: Hyperglycemia Diarrhea Hypotension Dehydration | Neither | BB: Without tender abdomen or fever we have multiple other causes for diarrhea, unlikely for diarrheal illness to cause sepsis especially with his hx of likely opiate withdrawal. Without source of infection would defer Sep bundles given more likely alternative diagnoses. |
| 009-S1-074 | 3.1 / Band 1 | Sepsis | Infection/Not Sepsis | Neither | AS: I would still do blood cultures with an LP. |
| 009-S1-003 | 3.1 / Band 1 | Not Infected | Infection/Not Sepsis | Neither | AP: **the band 1 rules out sepsis but not infection.** |
| 009-S1-103 | 3.5 / Band 1 | Not Infected | Other: polysubstance use, dehydration, agitation | Neither | SK: No change, no concern for infection, already confident prior to result. |
| 009-S4-167 | 3.6 / Band 1 | Not Infected | Infection/Not Sepsis | Neither | TP: On initial presentation the patient had abdominal pain. The history and exam given are not sufficient to say if there is a potential surgical/infectious etiology. I would not have order sepsis bundle treatment initially. His vital signs, WBC, and lactate are reassuring. The CT scan is probably the most important test here. **But having a band 1 result is helpful to exclude the possibility of sepsis early in this case.** |
| 009-S2-089 | 4.2 / Band 1 | Sepsis | Infection/Not Sepsis | Neither | JD: concern for infection developing into sepsis high given perforation |
| 009-S4-076 | 4.3 / Band 1 | Not Infected | Infection/Not Sepsis | Neither | CS: Altered elderly patient with abdominal pain and chronic smoldering infection. Middle of the road test result, not particularly useful in this case. |
| 009-S1-165 | 4.4 / Band 1 | Not Infected | Infection/Not Sepsis | Neither | EF: Did not change medical decision making. |
| 009-S2-057 | 4.5 / Band 1 | Infection/Not Sepsis | Infection/Not Sepsis | Neither | RB: Although this patient is Band 1, he has a fever and meets SIRS criteria so I would treat him as if he has sepsis unless there were a compelling reason not to (e.g., his fever could be explained by being covid + and his abdominal pain workup was negative). |
| 009-S4-117 | 4.7 / Band 1 | Not Infected | Other: Intracranial hemorrhage, hip fracture | Neither | AH: recent trauma could have affected results? |
| 009-S4-061 | 4.9 / Band 1 | Sepsis | Sepsis | Neither | EF: **Did not assist in medical decision making, still believe this is sepsis.** |
| 009-S4-061 | 4.9 / Band 1 | Sepsis | Sepsis | Neither | PF: intellisep says low risk but vitals and report of subjective chills makes me want to pursue sepsis treatment regardless, even if its probably covid. |
| 009-S4-007 | 7.1 / Band 3 | Sepsis | Other: The patient likely is presenting with acute renal failure and rhabdomyolysis. I do think the patient is critically ill and does have risk factors for an infection. I do not think sepsis is likely. I think the Intellisep is likely high due to the critical illness aspiratin pna is high as well. | Neither | RV: The results support critical illness not sepsis from an infection in this case. |
| 009-S5-174 | 9.3 / Band 3 | Sepsis | Infection/Not Sepsis | Neither | EV: This patient just doesn't seem septic. Clearly has an infection, but only slight tachycardia that can be attributed to fever to at least some degree, normal lactate. Could he progress to sepsis, yes, but right now I feel like the test strongly suggests sepsis but I'm just not seeing it from the case as presented.. |
| 009-S2-011 | 9.8 / Band 3 | Sepsis | Sepsis | Neither | JD: intermediate probability of sepsis - i would still treat for sepsis. |

**Example Case Vignette with details of information provided to the participant with and without the IntelliSep test result, and full list of questions asked.**

In each vignette:

1. The participant is asked to enter their name, their credentials, and if applicable, their medical specialty and level of training.


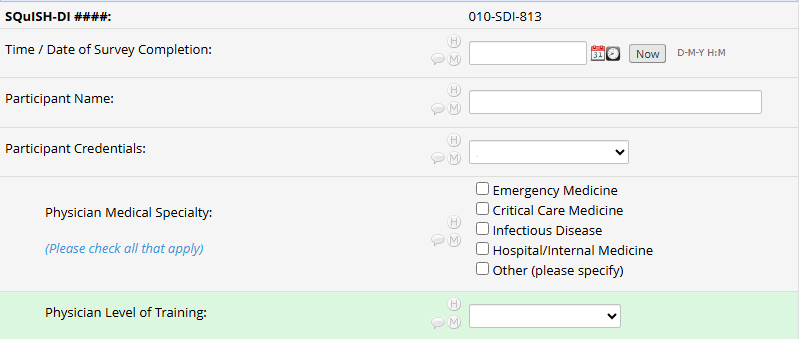


1.
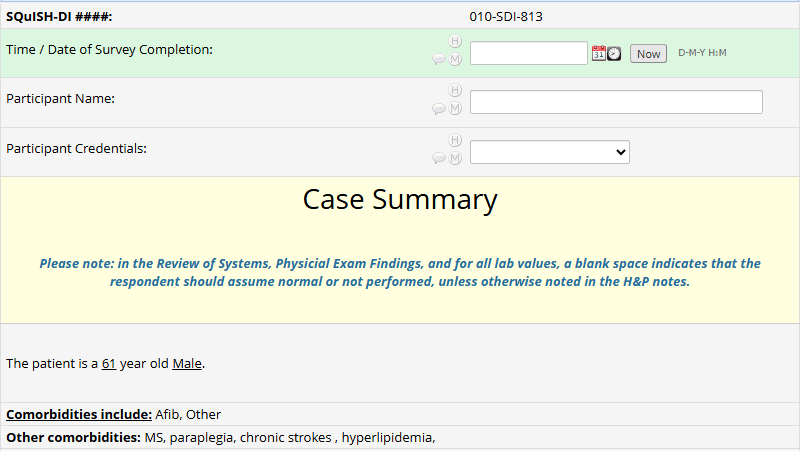
A case summary is presented, including data available early in the ED presentation (demographics, comorbidities, baseline Sequential Organ Failure Assessment scores, ED history & physical examination (H & P) notes, review of systems and exam findings, results of routine tests, e.g., chemistry, CBC, urinalysis, lactate, and troponin, as available).


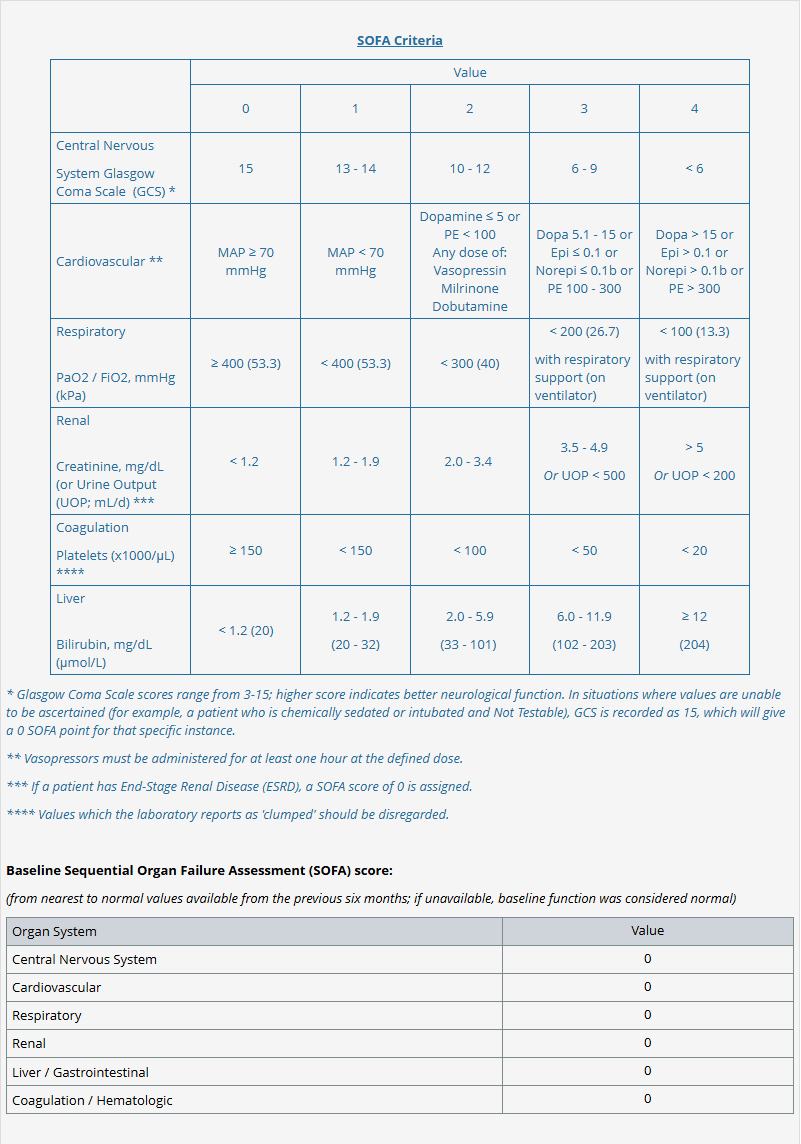


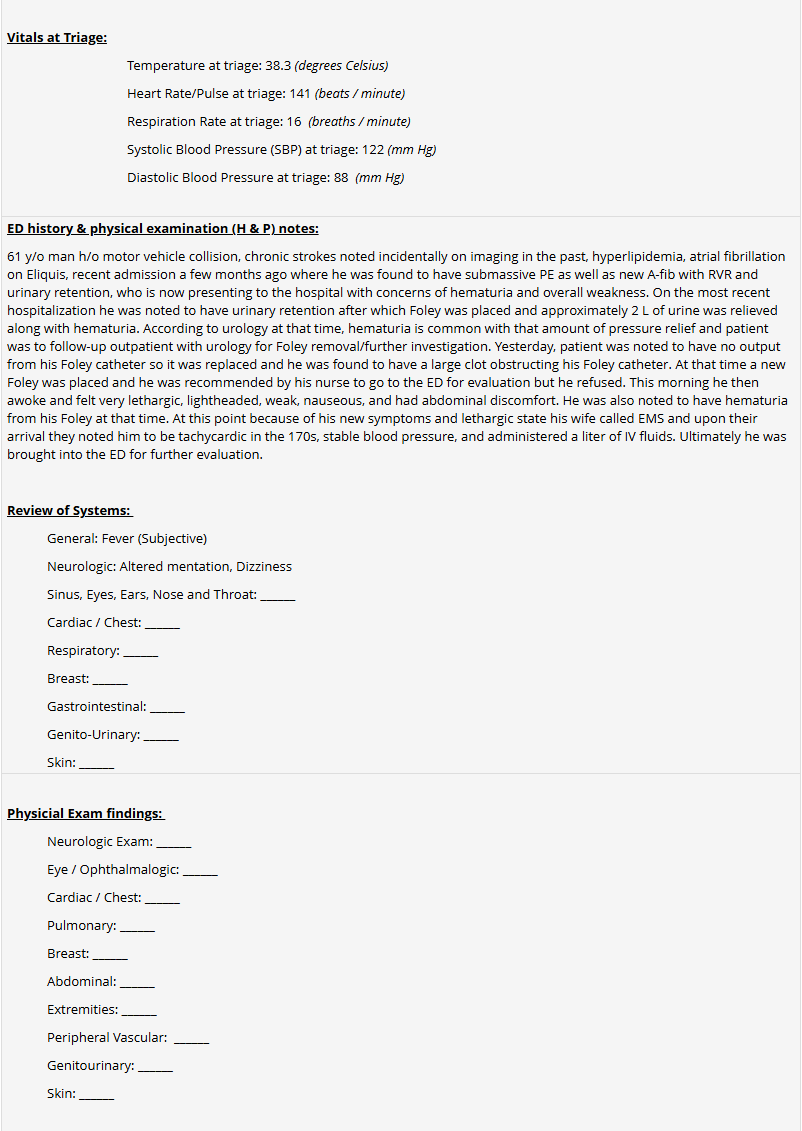


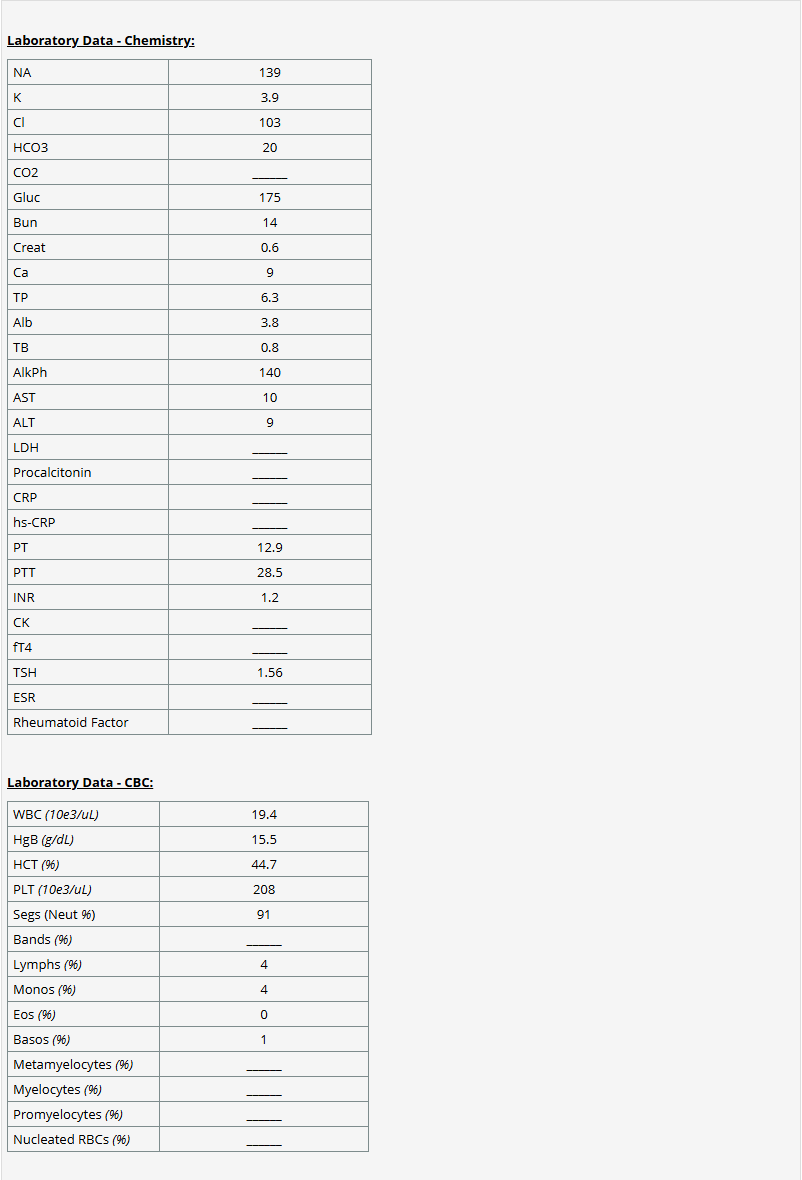

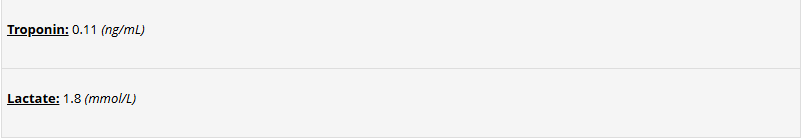


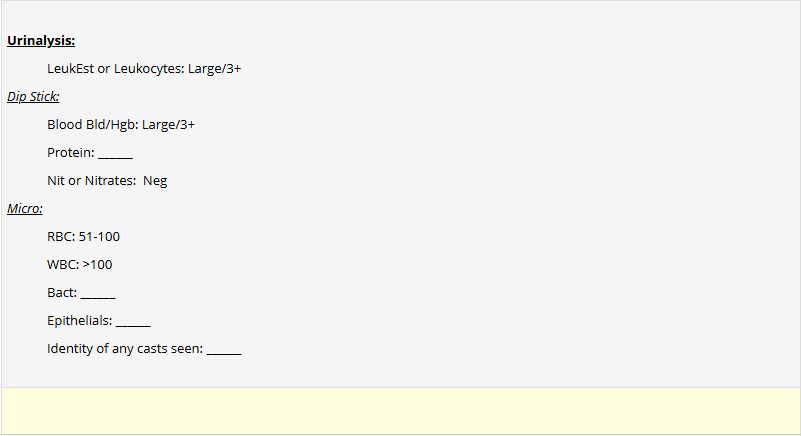


If applicable based on the stage of data collection, the IntelliSep Index and its corresponding Interpretation Band are presented as part of the case summary.


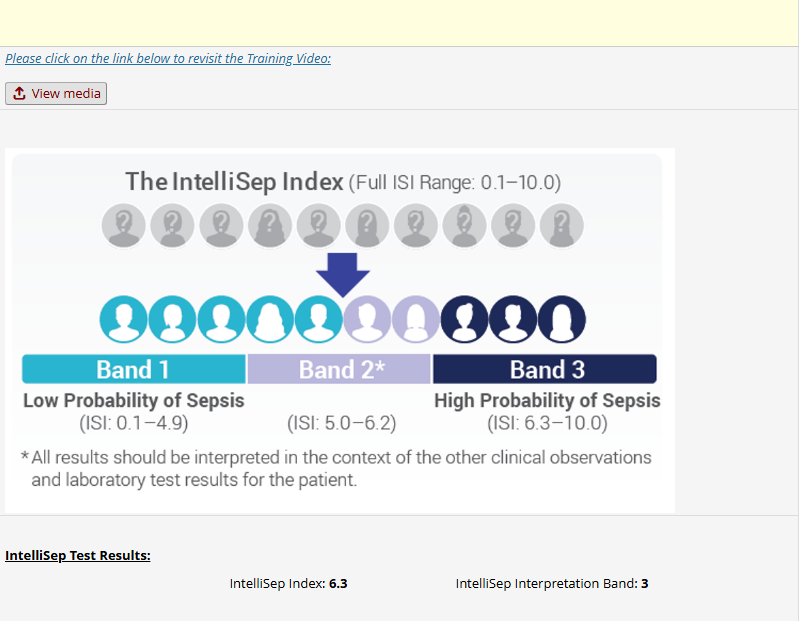


1. The participant is asked to choose their diagnosis (sepsis, infection, other), their level of confidence in the diagnosis, and whether they would expedite the SEP-1 care bundle for the patient.


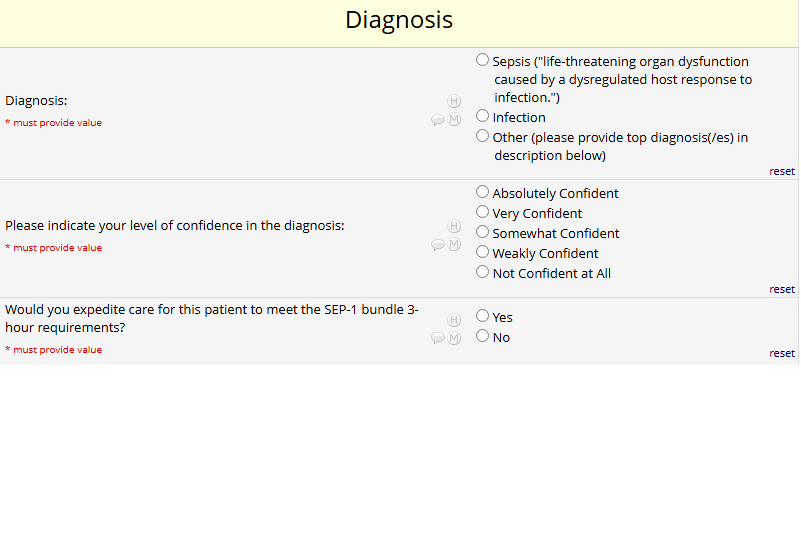


1. The participant is asked to indicate their decisions with regards to patient disposition, cultures, serology, and molecular diagnostics, ED interventions (IV antibiotics and type, antivirals, IV fluids, and vasoactive medications, as well as, advance diagnostics, imaging and surgical procedures).


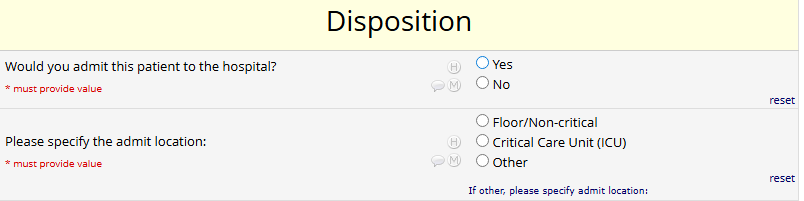


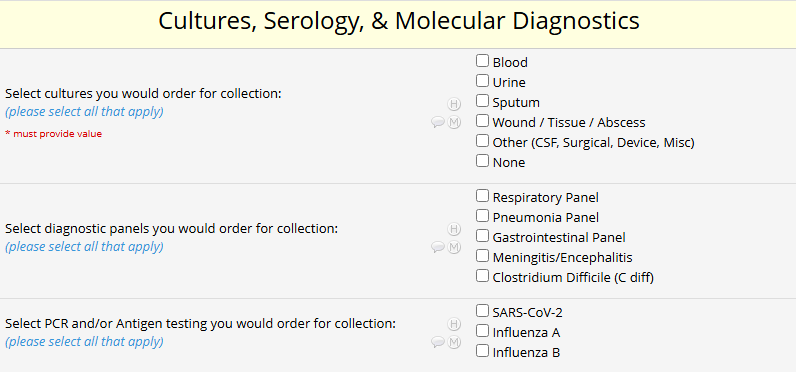


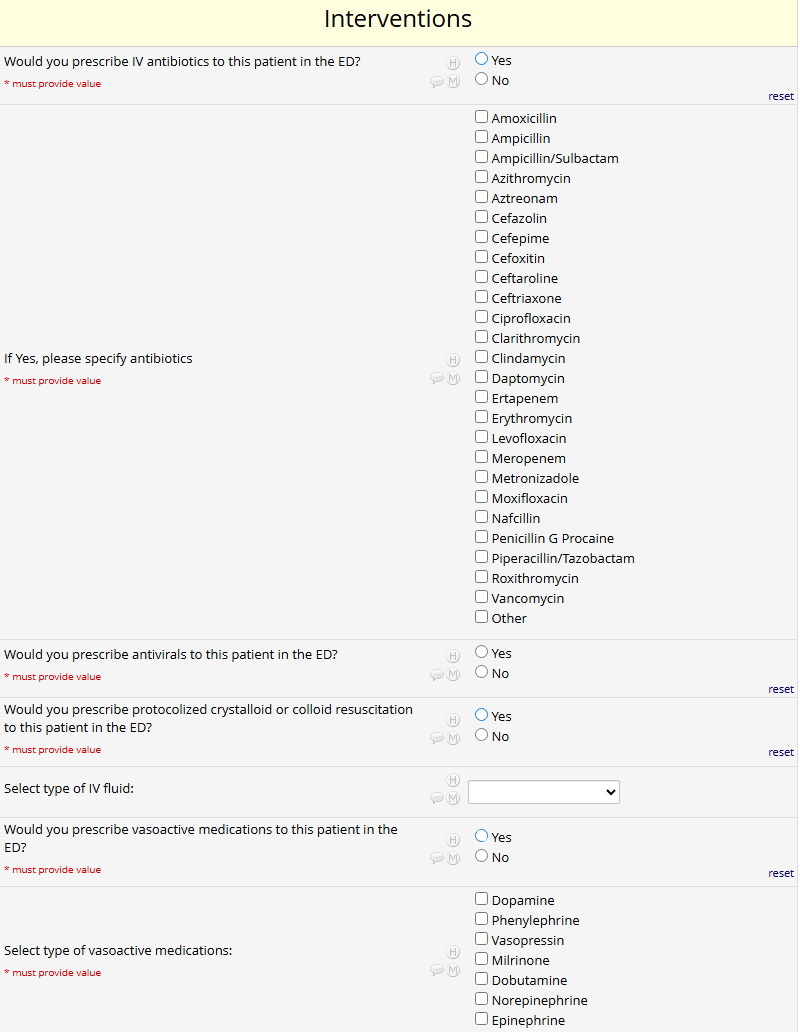


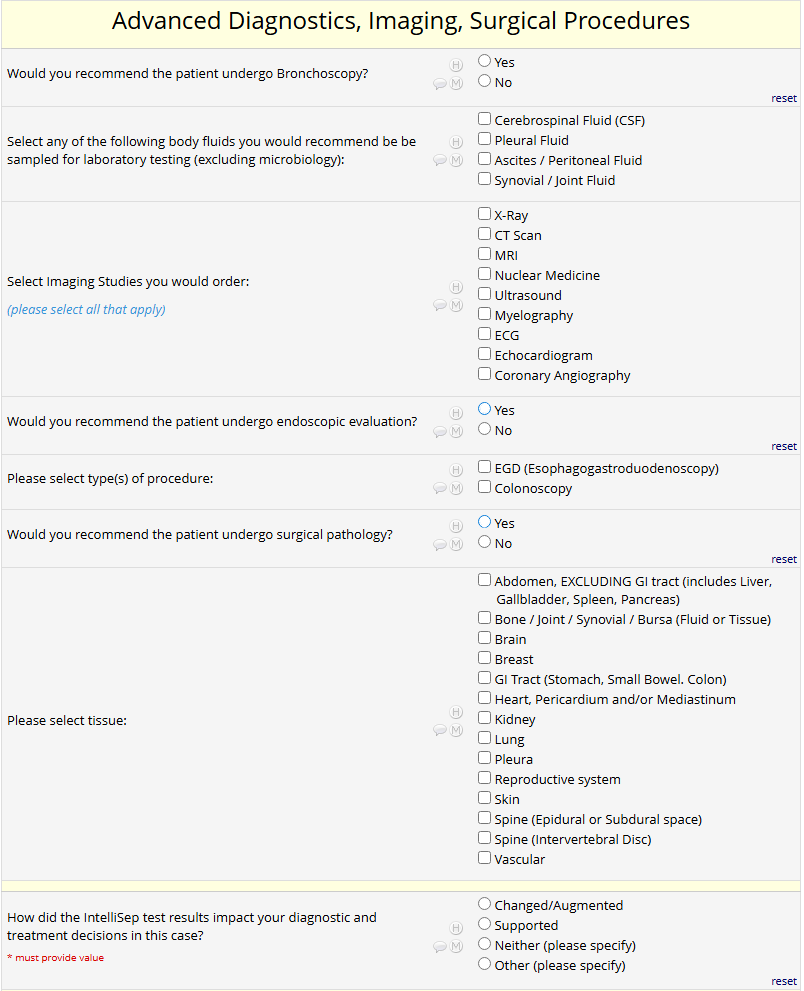


1. If the IntelliSep index was included as part of the case summary, the physician is asked to indicate how the IntelliSep test results impact their diagnostic and treatment decisions for the case.


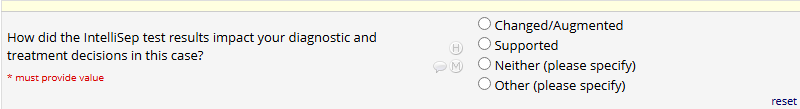

Supplement: Supplementary Material [file mmc1.docx]
